# Chicago Medical Society; Meeting of March 21st

**Published:** 1887-05

**Authors:** 


### SOCIETY REPORTS.

CHICAGO MEDICAL SOCIETY.

*Stated Meeting, March 21st, 1887.*—THE PRESIDENT, EDMUND J. DOERING, M.D., in the chair.

DR. JAMES I. TUCKER reported a case of "Herpes O.no-brachialis," which appeared in the April number of the JOURNAL AND EXAMINER.

DR. JOSEPH ZEISLER, in opening the discussion, thought cases of herpes zoster were comparatively rare here; in the last two years he had met with only one, affecting the fourth and fifth intercostal spaces of the right side. Although physiological and anatomical examinations have not yet proven the existence of true trophic fibres in the nerves, there can be no doubt that zoster must be considered as a tropho-neurotic disease. The case was especially interesting from the possibility of syphilis being the cause of the disorder, and from the large area involved.

DR. TUCKER said that one point in reference to herpes zoster, especially herpes zoster frontalis, is that chronic bronchitis is sure to follow it, and life generally terminates in that way.

PROFESSOR CHARLES WARRINGTON EARLE asked if the author had not found that these cases almost always require the same tonic treatment that neuralgias do, viz., the exhibition of quinine and iron, and a good, generous diet, before a cure can be effected.

DR. TUCKER replied that all the patients that he had seen suffering from the disease have been depleted, and certainly the remedies just mentioned would be needed.

PROFESSOR E. L. HOLMES reported a case of

CHANCRE OF THE EYELID.

Cases of chancre of the lids are of interest, not so much on account of their rareness, as of the difficulty in establishing a certain and early diagnosis, and the fact that suspicion even may not be awakened till quite late in the development. It is many years, probably twenty, since he had seen a case of primary specific sore on the genital organs. If he remembered correctly what he formerly observed, and what he learned from competent authorities, it is undoubtedly true that even an ulcer on the genital organs cannot always be easily diagnosticated. The swelling of the pre-auricular glands is quite often the result of simple abscess of the lids, especially if it is situated near the external angle. When, however, a circumscribed disease of the lids is accompanied by multiple pre-auricular and submaxillary glandular enlargement, suspicion becomes almost certainty. This has been the fact in four of the five cases which he had seen. In one case there was not sufficient swelling of the glands to attract either his attention or that of the patient.

Some months ago he had mentioned in this Society the case of a laborer in a rolling mill who received a severe burn on the side of the body from a "flash," and on the left upper lid from a drop of the molten slag. That small round burn became infected from some source unknown to the patient, and after some time presented the appearance of a true chancre with glandular swellings and characteristic eruptions. No evidence of a lesion could be found on the genital organs. Every symptom disappeared under energetic mercurial treatment.

Very recently Mr. P. M., 45 years of age, married, also a workman in a rolling mill, received a "flash," a portion of which struck the left upper lid and inflicted quite a severe burn of the integument. The conjunctiva was also slightly burned. At the end of a week the patient came to him with the lids much swollen, red and tender, with a hard, dry, thin and red incrustation as one sees on a superficial abrasion of the skin. The patient stated that he had rubbed the lids several times during the week with the back of his hand, which was more or less covered with grime and sand. He did this to relieve the irritation of the eye. There was nothing in the appearance of the lids or of the eye to excite suspicion of any complication. For ten days the eye was treated with mild lotions and ointments without the slightest benefit. On the appearance of pre-auricular and submaxillary swelling the patient was referred to Professor Hyde. Professor Hyde could simply suspect specific infection, but preferred to delay definite opinion for a few days. In a week or more the characteristic eruption appeared, with increased severity of the local symptoms. Large doses of blue mass and iodide of potassium relieved all the symptoms, local as well as general. The eye assumed its normal appearance in about three weeks.

PROFESSOR F. C. HOTZ said that as the author wished the discussion to be conducted on the line of experience of general practitioners, not specialists, he hardly felt that he was the right person to open it. As the author stated, these primary syphilitic ulcerations, chancres of the eyelids, are of very rare occurrence, and in a long experience of private and hospital practice in this country and Europe he could remember but one case coming under his observation of a primary syphilitic sore, and it was located on the conjunctival surface of the upper lid—a most curious place for the infection. A patient came to him about two years ago with the lid tender and very much swollen, and on turning it he saw a large ulcer with all the typical appearance of chancre of the prepuce. There was no doubt of its syphilitic character, but it puzzled him as to how the infection could have taken place there. On questioning the patient he learned that four or five weeks previously he got a little cinder or something in his eye, and his room-mate turned the upper lid, saw the foreign substance, and removed it with his tongue. Further questioning brought out the fact that this man was syphilitic.

DR. JOSEPH ZEISLER mentioned the possibility of contamination by kissing and by instruments. The most peculiar cases of chancre, in regard to the locality, had been related by Deloir in his "Discours sur le Syphilis," some of which could only be explained by certain French habits.

PROFESSOR WILLIAM T. BELFIELD said, in reference to one point raised by Professor Holmes, viz., infection without ulceration, he thought it was a well known fact that it has frequently occurred. There is nothing about syphilitic infection that implies ulceration. The morbid material is introduced into the skin and occasions a certain amount of inflammation at the point of infection. Whether that results in ulceration depends on the nutrition the parts receive; if they are well nourished there need be no ulcer, but as a rule some of the cells that are formed die and ulceration is the result. Occasionally the inflammation is so slight that neither ulceration nor even induration is produced locally; since syphilis is undeniably inoculated—in exceptional cases—without the production of any features of the hard chancre. Nor is the syphilitic the only virus whose local effects are thus variable; thus, the virus of charbon, or "wool sorters' disease" while usually producing a malignant pustule at the point of inoculation, may be communicated without any local ulceration—indeed by simple inhalation.

PROFESSOR D. A. K. STEELE read a paper entitled

### INTUSSUSCEPTION IN INFANTS.

Intussusception or invagination of the upper into the lower part of the intestine, embraces three layers of the bowel which include all the coats of the intestines. The diagnosis of intussusception in an infant is usually an easy matter; a baby previously in good health is taken with sudden violent vomiting, with loud cries and evidence of abdominal pain and uneasiness occurring at frequent irregular intervals, accompanied with severe straining and the passage at first of fecal matter, then a mucous tinged with blood, and later of blood alone in considerable quantities. Thirst, anxious expression and collapse are marked symptoms. A distinct sausage shaped tumor can frequently be felt at the seat of the obstruction in the abdomen.

Treatment may be divided into medicinal, mechanical and operative. The medicinal plan is palliative but rarely curative.

The author described the details of the mechanical plan of treatment as follows: Having clearly established a diagnosis of intussusception and previously given an opiate to quiet all peristalsis, he anæsthetizes the child with chloroform and then carries a rectal tube or large catheter about six inches within the anus. He attaches to this tube a flexible rubber tube two or three feet long attached to a small glass funnel. Into this funnel are poured alternately solutions of soda bi-carbonate and tartaric acid, thus generating carbonic acid gas and rapidly and safely inflating the large intestine. Three drachms of each will generate enough gas to completely distend the colon of a child. If this pressure is not sufficient to relieve the obstructions, he attaches an ordinary double bulb syringe and inflates with air about as much as he thinks the bowel will stand; while the abdomen is being carefully manipulated by an assistant, he patiently, persistently and repeatedly employs these measures coupled with massage, abdominal taxis, inversion of child, etc., from twenty-four to forty-eight hours, when, if there is no relief he advises laparotomy. In case the obstruction is at the ileo-cæcal valve and is irreducible and an artificial anus cannot be readily formed, an ileo-colotony or implantation of the divided ileum above the obstruction into a slit-like orifice in the colon below the obstruction would be justifiable procedure. Senn's recent experiments on animals prove that it can be done with success. Several cases were reported which had been successfully treated by the mechanical method.

PROFESSOR C. W. EARLE, in opening the discussion, said that Professor Steele had presented the symptoms of this terrible difficulty so fully that there is little more to be said. He was glad that the author dwelt upon the symptom of constriction of part of the bowel, which is probably one of the chief causes for this accident. He must take issue with him in regard to the ease with which he diagnosticates some of these cases. Professor Steele's experience has been very different from his own when he is able to say that the diagnosis of intussusception in a child is made out without much difficulty. In a number of cases he has seen there has been an absence of all the symptoms which the author has narrated. He remembered a case which occurred several years ago in an outside village, in which he made the post mortem examination and found a very distinct intussusception. In that case we could get none of the symptoms spoken of; there was no tenesmus, no bloody discharge, no protrusion of the invaginated portion of the intestine, no sausage-shaped tumor of the abdomen, and yet the child had intussusception, probably from eating a large amount of cherries as a number of cherry pits were found in the bowel at the autopsy.

As a medical man it might be expected that he would oppose a surgical procedure, but the medical treatment of this difficulty is so absolutely hopeless that it seems to him if laparotomy offers anything at all we should be ready to accept any procedure the surgeons have to offer. However, the results of Professsor Steele's process of treatment by inversion, and shaking and filling the bowel are really better than those of surgeons up to this time. It is a well known fact that laparotomy for the relief of intussusception is followed by greater mortality than laparotomy for any other cause. But if the diagnosis is well made out, and particularly if the pulse is rapid and the child shows profound symptoms which point unmistakably to this difficulty, it seemed to him that we are hardly justified in waiting three days; he thought at the end of twenty-four hours we should cease to try the ordinary methods and proceed to do the operation.

PROFESSOR F. E. WAXHAM said he agreed with Professor Earle in regard to the difficulty of making the diagnosis in cases of intussusception, especially in infants. In many cases he thought the diagnosis very difficult, and sometimes a certain and absolute diagnosis is almost impossible. This is true for the reason that all the symptoms that have been enumerated as characteristic of the disease are not present in every case. The tumor is as frequently absent as present; if a small amount of intestine is involved in the intussusception it is impossible to detect it. Again, the bloody discharges are frequently absent, and the protrusion of the intestine from the anus, which is given as a prominent symptom, is often absent. Indeed, in many cases the incessant vomiting, obstinate constipation and the tenesmus are the only symptoms. He would refer to the differential diagnosis of intussusception: fecal impaction occasionally presents symptoms analogous to intussusception. Impaction is rare in infancy, but is more frequently observed in the adult and in the older children. It may give rise to a tumor, vomiting, tenesmus, tympanites and even protrusion of the intestine from the anus. These symptoms closely resemble those of intussusception, but in impaction the vomiting is not usually as obstinate and large; copious enemas will cause the disappearance of the impaction. Again, in impaction we do not get the rapid and great prostration that is observed in intussusception. Cholera infantum may be mistaken for intussusception, for we have the same rapid and great prostration and collapse, we have the incessant vomiting, but in cholera infantum we have the large, copious watery passages instead of the obstinate constipation followed by the bloody discharges. Dysentery presents symptoms closely resembling intussusception in many cases; we have vomiting, tenesmus, frequent straining, bloody passages and not unfrequently protrusion of the intestine from the anus, but in dysentery the vomiting is not so obstinate nor the prostration so great. Typhlitis may present symptoms very closely resembling intussusception; as the result of inflammation at the ileo-cæcal valve we will have vomiting, intestinal pain, tympanites—obstinate constipation which it is impossible oftentimes to overcome. The diagnosis in these cases is very difficult, and he said he knew of one case of typhlitis where several surgeons thought seriously of operating for a suspected intussusception.

But in typhlitis the tumor, which results from inflammation and infiltration is in the right side, while in intussusception the tumor when observed is in the left iliac region or in the transverse colon, for although the intussusception takes place at the ileo-cæcal valve, if the tumor is large enough to be observed, the smaller intestine rolls in the larger until the tumor appears in the left side instead of the right. In typhlitis we have a history of acute inflammation, not so in case of intussusception. Peritonitis will also frequently give rise to symptoms resembling those of intussusception; we have frequent vomiting, obstinate constipation, abdominal pain and tympanites. He remembered a case that was treated for several days for intussusception, and not until the administration of morphia and lime water was the vomiting overcome and a correct diagnosis made. The vomiting could not have been relieved by medication in a case of intussusception. In peritonitis we usually have the gradual onset of the disease, more sudden in case of intussusception. In peritonitis we have the symptoms of acute inflammation existing for several days before we get symptoms of obstruction. In intussusception the symptoms of obstruction and shock appear early. In peritonitis, although the constipation is often obstinate, yet the vomiting is not as frequent and uncontrollable as in intussusception, nor do we have the rapid prostration in the former disease that is so characteristic of the latter. There are other forms of obstruction, such as the twisting of the intestine upon itself, pressure upon the intestine by a band of lymph, or strangulation of the intestine in a congenital opening in the diaphragm, any of which will give rise to symptoms exactly corresponding to intussusception.

In regard to the treatment, little remained to be said. Not infrequently, when an intussusception is suspected, the cases are treated with physic, castor oil, croton oil, or quicksilver, in order to prove the diagnosis. This treatment should be mentioned only to be condemned. No remedies should be given that will increase the peristaltic contraction, but opiates given to prevent it. Quicksilver may be used with benefit in those cases where the invagination is low down in the bowel, or where the intussusception is of such extent that it reaches into the lower bowel, or perhaps near the anus. In such cases the child should he inverted and the quicksilver used per rectum, and the pressure and weight of the quicksilver will assist greatly in the restoration of the invaginated mass. He fully agreed in the recommendation of laparotomy in cases where mechanical measures fail. When we have faithfully but unsuccessfully tried medicinal and mechanical measures, he thought we should resort to laparotomy, providing a positive diagnosis can be made, and we should not delay too long, for the longer the operation is delayed the less the chances are of recovery.

PROFESSOR A. E. HOADLEY wished to make one suggestion as to the surgical treatment of such cases. The author, on going into the surgical treatment thoroughly, had left the idea, he thought, that about all that can be done for cases of acute invagination of the intestine is to unfold the invaginated intestine, and if this cannot be done, it must be excised and an artificial anus made. Of course he does not advise the resection of the invagination, but advises an artificial anus as a temporary means. Where invagination is acute it obstructs the lumen of the intestine, and all the urgent symptoms are consequent on the obstruction of the bowels, not because the tissues are liable to ulceration or sloughing, provided that the tension above the invagination is promptly relieved. If the tension above the stricture is relieved the tissues at the stricture can take care of themselves, the strangulation will be immediately relieved in a measure, so that the circulation can be carried on at the seat of invagination. Relief of tension controls peristalsis, and no further invagination will take place, congestion will be relieved and the tissues saved. There has been an operation suggested for that purpose which is applicable in all cases where to unfold the invagination is more dangerous than to leave it alone or excise and establish an artificial anus. The operation is to bring that portion of the bowel above the invagination to that immediately below and there make an opening in each, stitch the openings together, and thus form an outlet for the distended intestine above. In case the invaginated portion loses its vitality there is no objection to its sloughing, as the slough can readily pass down, as the gut is not closed above. In his own practice he had seen two cases of recovery from intussusception, in which there was no doubt about the diagnosis; in one case a sausage-shaped tumor could be felt across the median line, with all the acute symptoms except, perhaps, the bloody discharges. The invaginations in these cases were of the colon, and not ileo-cæcal, and the lumen of the gut was not completely closed. There was no particular distension, but there was a large and painful tumor, with tenesmus and diarrhœa. In one case, in which the diagnosis was confirmed by several physicians, the tumor remained for more than a year, and at any time, if pressure was made over it, there would be tenesmus of the bowel; yet the child, who is now 8 years old, fully recovered. He then had a patient under treatment in whom two and a half months ago there was probably intussusception of the colon. A painful tumor presented on the left side which could be plainly felt from day to day, associated with all the irritable symptoms of the bowels, but the invagination did not occlude the lumen of the bowel, therefore the most distressing symptoms were absent. The tumor gradually grew smaller, less tender, less painful, and the child became free from the irritation caused by that invagination.

Dr. J. FRANK asked if, when the invagination is situated above the ileo-cæcal valve, water injected would pass the valve itself? He hardly thought the small amounts that the author spoke of having injected would have reduced the invagination. Dr. Frank was not opposed to giving cathartics in cases where they might do good, and thought sometimes they do not do as much harm as an operation. The intestines are held up by mesenteries, and if the principal part of the intestine cannot invaginate itself where it is held fast it cannot come down. A cathartic produces peristalsis which is always towards the rectum, and if the part is held up by the mesentery, the peristaltic motion will not bring it down any further, and yet will relieve the intussusception. He thought many cases are cured by cathartics. He had a case some time ago where the child was pulseless and cold, the eyes turned in the sockets, and there was tenesmus and vomiting. After trying nearly everything a large dose of calomel was given, and as soon as there was a movement of the bowels the child was all right.

PROFESSOR STEELE, in closing the discussion, said, in regard to Professor Earle's query as to the ease of making the diagnosis: the diagnosis is not always an easy matter. In the first case reported the diagnosis was easy, because the case presented the symptoms laid down in the books as nearly as could be: there were vomiting, tenesmus, bloody stools, absolute constipation and a lozenge-shaped tumor—but that is the only case of the four reported in which there were all the symptoms; in two there were no tumors, and in one the tumor was indistinct and irregular in outline. There was no pain in that case. In none of the cases was the diagnosis verified by post-mortem examination. There is an element of doubt as regards the diagnosis, but it had been formed in each case, after careful examination by competent physicians, and was considered fairly certain. In regard to the relative safety of ether and chloroform in children, he could not give the statistics, but the administration of chloroform to children is a very safe procedure as compared with its administration to adults. In regard to the differential diagnosis, Professor Waxham presented all the points very fully. In a paper of this character he did not wish to go into that subject. In regard to the operation of cutting the bowel above and below the point of obstruction, that he alluded to as an operation having been done experimentally on animals with success by Professor Senn, of Milwaukee. So far as he knew, there are no statistics showing that it has been done on man. The case of obstruction occurring in a child, with painful tumor, spoken of by Professor Hoadley, was a case of chronic obstruction in which the lumen was not perfectly occluded, and those cases are not dangerous. So long as the lumen is not absolutely interfered with the processes of nutrition go on. Cases of chronic obstruction were not considered in the paper; he spoke only of acute obstruction due to intussusception. Water passes the ileo-cæcal valve by dilatation from over-distension. He had seen the experiment tried on animals; by opening the abdomen and forcibly injecting water, the colon would become distended to its utmost capacity, and by-and-by the tissues would begin to yield, the valve would become incompetent and the water would pass. The quantity of water used for injections was smaller than if the obstruction had been higher up. The case of acute obstruction mentioned by Dr. Frank, that was relieved by a large dose of calomel, was probably acute enteritis, such a case as would have been relieved by bleeding many years ago. When we are called to these cases of acute intestinal obstruction where there is a doubt as to its cause, we should take into consideration all the factors; the age of the child, the sex, the suddenness of the onset, the presence of a majority of symptoms that are said to be present in intussusception, and then make a thorough, complete and systematic palpation of the abdomen, aided, if necessary, by the distension of the colon with carbonic acid gas. By the alternate injection of an acid and alkaline solution into the rectum, one can distend the colon so that it can be mapped out perfectly, and the location of the obstruction be determined.

PROFESSOR ELBERT WING exhibited some pathological specimens, and said that these specimens were obtained at the County Hospital and had been brought immediately, because specimens are more interesting when they are fresh. The case came to the dead-house without any history except that the man was known to have had some trouble with his heart. The internes were careless, and no careful record was kept. The man was about 60 years old and very fat. There was no fluid in the abdominal cavity. The pericardium contained perhaps 30 cu. cm. of clear serous fluid, and the heart was considerably larger than the man's fist, with one or two of the so-called "milk-spots" on the surface. The right auricle was distended with dark, partly clotted blood, the right ventricle contained a very small quantity of fluid blood and a small clot; the left auricle and ventricle were empty. Upon section the heart showed no lesion of the valves; there was slight atheroma at the base of the aorta. The endocardium showed the streaks and bands of grayish discoloration which are taken as evidence of chronic endocarditis; there was also, over the surface of the endocardium, the mottled appearance of myocarditis with fatty degeneration. The left pleural cavity was empty and without adhesions. The surface of the lung presented a few spots of emphysema, and upon section the bronchial tubes were found extremely congested and lined with a thick coat of mucus throughout the bronchial tree. On the right side there was an adhesion along the median line, and the pleural cavity contained 3,700 cu. cm. of a clear serous fluid, slightly reddish, containing no shreds and no plaques of fibrin. The entire costal pleura of this side was much thickened, of a slightly velvety appearance and bright red color, easily separated from the underlying tissue and easily torn in manipulation. The deposit of fibrin could be removed only by considerable force, and the pleural surface then showed manifold small red dots, resembling the mouths of severed capillaries. Completely covering the surface of the right lung there was a film of lymph which varied in thickness from that of a heavy sheet of blotting-paper to 1 or 2 millimetres. Over the lower lobe this had taken on organization, and contained bands of connective tissue. Throughout the extent of the visceral layer of the pleura it could be removed, leaving the surface smooth and glistening. The lung on the cut surfaces was of a steel-gray, mottled with spots of pigment discoloration. It was completely devoid of air, and a piece cut out from it and thrown into the water would sink. Upon the superior border of the spleen there was a small plaque in which the connective tissue of the capsule was largely increased. The cut surface of the organ presented a mahogany color and as a whole was quite firm, verifying the diagnosis of what we would expect to find in such cases, chronic passive hyperæmia of the spleen causing an increase of the connective tissue. The kidneys were hardly enlarged at all, the capsule was not thickened and was easily removed. Upon section the cut surfaces of each of the organs was a very dark red color, the vessels all full of dark-colored blood, and the organs slightly firmer than normal. The left lobe of the liver presented a peculiar appearance. It looked and felt a little granular, which seemed to be due to a wrinkling of the capsule. There was a similar appearance along the anterior border, and near the line of the suspensory ligament. That large spot was triangular upon the surface, say 5 cm. along each side, and extended into the organ in a pyramidal shape, the apex being near the hylus of the organ. The line of demarcation between the wrinkled and the unchanged surface of the organ was sharply defined. On section these portions of the organ presented the characteristic appearance of advanced simple atrophy, a dark brown slate-color, the lobule distinct but very much diminished in size. That portion of the organ not described was in the condition known as "nutmeg liver." The omentum was in a condition rarely seen. It was 25x12x1½ cm., somewhat lobulated, and along the anterior surface there were broad radiating bands of fibrous tissue. The organ as a whole was very firm and solid, and on section little lobules of fatty tissue could be isolated. The condition was that of marked hypertrophy of the fatty tissue of the organ, together with a great increase of the connective tissue, the latter due to chronic passive hyperæmia, the former part of the general marked increase of adipose tissue throughout the body. There was also considerable œdema of the right leg, but no thrombosis in the iliac vein. Examination of the veins outside of the abdominal cavities was not permitted. With the conditions described as a basis, the diagnosis was: chronic endo-myocarditis with fatty degeneration; chronic pleuritis (costal) with effusion, atalectasis from compression, right side; chronic passive hyperæmia of spleen, kidneys and liver, with added circumscribed simple atrophy of the latter; hypertrophy and chronic passive hyperæmia of the omentum; chronic venus thrombosis with œdema of the right leg.

PROFESSOR T. BELFIELD read a paper on

### DIGITAL EXPLORATION OF THE KIDNEY, WITH REPORT OF THREE CASES.

Of some 250 cases of nephrectomy heretofore reported, about 44 per centum have died from the effects of the operation. The chief causes of death have been:

1. Shock (42 per centum of the fatal cases).
2. Uræmia (14 per centum).
3. Peritonitis (21 per centum).

These three factors were thus responsible for 77 per centum of all deaths. Improvement in the results of renal surgery seems, therefore, to require means for the avoidance of these three great dangers.

The chances of fatal shock are decreased by early operation, before the patient is exhausted and the damage to kidney-tissue extensive through long illness. This self-evident proposition is illustrated by the fact that, while about 18 per centum of all (250) nephrectomies have died of shock, yet of 24 nephrotomies of fairly healthy kidneys removed for wounds of loin, ureter, for mobility, etc., not a single death occurred from shock.

The greatest obstacle to the early surgical treatment of renal diseases has been *faulty diagnosis.* Such cases are usually treated as lumbago, sciatica, hip joint disease, and especially cystitis, for a long time before the true seat of the lesion is detected or even suspected. Since it is not always remembered that pyelitis produces symptoms identical with those commonly ascribed to cystitis, the former is not suspected, and a diagnosis of cystitis is made; and as cystitis is still too generally regarded as an entity—a disease instead of a mere symptom requiring explanation—the patient is treated for "chronic cystitis." Finally, months or years later, a lumbar swelling or other symptom reveals the renal origin of the difficulty. Meanwhile the patient has become so enfeebled that he succumbs to the shock of an operation that could have been safely borne at an earlier period. Early exploration of the kidney—a comparatively slight operation—has, by drainage of the pelvis, in many cases relieved a difficulty which at a later stage would have required nephrectomy, a formidable operation; moreover, a preliminary drainage has been shown to be a valuable factor in diminishing mortality from nephrectomy. Thus, of seventy-three nephrectomies for suppurative lesions collected by Gross, twelve were made after previous exploration of the kidney, with but one death; of the remaining sixty-one —without previous nephrotomy—thirty-one died.

Uræmia rarely occurs after any renal operation provided the second kidney be present and in healthy condition. While commonly associated with nephrectomy, one of the annexed cases shows that uræmia may be inevitable after simple nephrotomy; hence a necessary preliminary to any renal operation should be an attempt to ascertain the functional condition of the opposite kidney. In the female this can often be accomplished by cauterizing the ureter, after Pawlik's method; in the male probably the best, though still an uncertain means, is Silbermann's instrument for compressing one ureter. The statement that the abdominal incision, by permitting digital examination of the opposite kidney, obviates the dangers of uræmia, is fallacious; for in many cases kidneys which are indistinguishable by the finger, or even by the eye, from normal organs, are extensively invaded by disease—inflammatory, tuberculous or calculous. The kidneys exhibited by Professor Steele are signal examples—normal in size, contour and consistence, yet each extensively tuberculous. Palpation through an abdominal incision could have detected nothing abnormal in either of those kidneys.

The following cases illustrate several of the principles above enunciated:

S. M., widow, 26 years old, delicate since childhood; has had dyspepsia for years; for a year past has suffered from headache, pain in back and left thigh; menstrual intervals shortened to two or three weeks. Was variously treated for lumbago, sciatica and ovarian disease. In March, 1886, urination was unduly frequent and sometimes painful; urine contained pus and blood; was treated for cystitis.

On admission she was emaciated and feeble; urination every half-hour or oftener and painful; had frequent pain in lumbar region, often shooting down to left thigh; and required morphine constantly. Urine acid, depositing blood, pus, and occasional rosettes of large uric acid crystals.

Diagnosis, calculous pyelitis, probably limited to left side; right ureter catheterized and urine appeared normal.

November 29th, 1886, the kidney was exposed and the pelvis explored; mucous membrane rough and ulcerated, clots of pus evacuated; wound in kidney packed with gauze.

Except for persistent vomiting for a few days, recovery was without notable incident. The renal fistula was closed on the twenty-first day; patient has since been free from all her former symptoms and is regaining flesh and strength.

G. F., 30 years old, was knocked down by a cable car November 28th, receiving a lacerated wound of right hand and various contusions. The hand was dressed, and patient presented no notable symptoms until December 11th, when often straining at stool he passed a large quantity of blood with the urine and had severe pain and tenderness over the right kidney, with some temperature; these symptoms persisted in spite of rest, ergot, gallic acid and opium; the right loin while not swollen, felt boggy. December 22nd, the patient having experieuced several irregular chills and sweats during the previous day, a lumbar incision was made down to the right kidney. The peri-renal tissues were found closely adherent, extravasated blood was seen under the capsule and a rent one and a quartes inches long was found in the posterior surface of the kidney extending into the pelvis; about half an ounce of a watery fluid lay behind the kidney. A large drainage tube was inserted. The temperature remained high for several days, after which recovery rapidly ensued, and January 14th the patient was discharged well; the wound entirely healed.

C. L., 48 years old, farmer, began some two and a half years ago to suffer from slight pain and stiffness in the loins; later, pus appeared in the urine; was treated for Bright's disease. About a year ago urination became frequent and sometimes painful; was supposed to have cystitis. Has lost flesh; his rest has been much disturbed by frequent calls to urinate. Six months ago he passed two small calculi after some renal colic on left side; since that time has had slight colic on left side several times, followed by expulsion of clumps of pus but no calculi.

Diagnosis, tuberculous or calculous pyelitis, probably former; bacilli were found in one examination of pus, and the lower organs were normal.

Exploration, January 26th. The kidney (left) was considerably enlarged; no calculus could be detected with finger or needle. Incision through kidney showed the pelvis considerably dilated, its orifice narrowed by a thickening of upper part of ureter; mucous membrane ulcerated in several places; the pyelitis evidently tuberculous.

Nothing of note occurred until the fifth day, when the urine became scanty and tinged with blood; soon the usual evidences of uræmia became manifest, and death ensued from this cause on the eleventh day. A partial autopsy showed that the right kidney was extensively, the left slightly tuberculous; bladder and prostate normal. Evidently the right kidney, which had never caused pain, had been first diseased; the function of the left, which had latterly been performing most of the renal excretion, had been arrested, not by the slight incision, but by the congestion which followed the removal of the accustomed pressure in the dilated pelvis. Similarly uræmia has followed the sudden evacuation of a habitually distended bladder.

To summarize: 1. Surgical affections of the kidney and its pelvis are frequently masked under symptoms of cystitis, lumbago, hip-joint diseases, etc.

2. Digital exploration of the kidney and its pelvis often affords the only available means for accurate diagnosis in the early stages.

3. When made with due precautions, this operation is almost devoid of danger; while saving the kidney, it may accomplish all that a dangerous nephrectomy could offer at a later stage; and if it fails to cure, it materially increases the chances of recovery from a subsequent nephrectomy.

4. An investigation into the functional condition of the opposite kidney should precede an exploratory incision into the pelvis.

